# A Specific Interface between Integrin Transmembrane Helices and Affinity for Ligand

**DOI:** 10.1371/journal.pbio.0020153

**Published:** 2004-06-15

**Authors:** Bing-Hao Luo, Timothy A Springer, Junichi Takagi

**Affiliations:** **1**Center for Blood Research (CBR) Institute for Biomedical Research and Department of Pathology, Harvard Medical SchoolBoston, MassachusettsUnited States of America

## Abstract

Conformational communication across the plasma membrane between the extracellular and intracellular domains of integrins is beginning to be defined by structural work on both domains. However, the role of the α and β subunit transmembrane domains and the nature of signal transmission through these domains have been elusive. Disulfide bond scanning of the exofacial portions of the integrin α_IIβ_ and β_3_ transmembrane domains reveals a specific heterodimerization interface in the resting receptor. This interface is lost rather than rearranged upon activation of the receptor by cytoplasmic mutations of the α subunit that mimic physiologic inside-out activation, demonstrating a link between activation of the extracellular domain and lateral separation of transmembrane helices. Introduction of disulfide bridges to prevent or reverse separation abolishes the activating effect of cytoplasmic mutations, confirming transmembrane domain separation but not hinging or piston-like motions as the mechanism of transmembrane signaling by integrins.

## Abstract 2

Integrin receptors mediate cell-matrix interactions by altering the conformation of their intra- and extra- cellular domains, a process mediated by lateral separation of the transmembrane helices

## Introduction

Integrins are major metazoan cell adhesion receptors that have the distinctive property of transducing signals across the plasma membrane in both directions. Intracellular binding of cytoskeletal components to integrin cytoplasmic domains activates the ligand binding competency of the extracellular domain (inside-out signaling). Furthermore, ligand binding to integrin extracellular domains is coupled to alterations in cytoplasmic domains that are linked to downstream signaling (outside-in signaling). The three-dimensional architecture of integrin extracellular domains as well as their rearrangement in activation have been revealed by crystal, nuclear magnetic resonance (NMR), and electron microscopic methods (Xiong et al. 2001, 2002; Adair and Yeager 2002; Beglova et al. 2002; Takagi et al. 2002, 2003). NMR structures of integrin α and β subunit cytoplasmic tails (Vinogradova et al. 2000, 2002; Ulmer et al. 2001; Weljie et al. 2002) and a crystal structure of the β subunit tail in complex with the cytoskeletal protein talin (Garcia-Alvarez et al. 2003) yield structural insights. It is generally accepted that an intersubunit association at the cytoplasmic domain maintains integrins in the low-affinity state (Hughes et al. 1996); however, specific heterodimeric interaction between the isolated cytoplasmic domains in solution is sometimes not observed (R. Li et al. 2001; Ulmer et al. 2001), and when observed the reported structures differ (Vinogradova et al. 2002; Weljie et al. 2002). The dynamic nature of cytoplasmic intersubunit association was revealed using live cell imaging (Kim et al. 2003), which demonstrated upon integrin activation a decrease in fluorescent resonance energy transfer between yellow fluorescent protein and cyan fluorescent protein tags fused to the C-termini of the integrin α and β subunit cytoplasmic domains. This finding demonstrated separation of the cytoplasmic domains; however, whether signal transmission through integrin transmembrane (TM) domains involves hinging or pistoning motions or lateral separation in the plane of the membrane has yet to be definitively established (Hughes et al. 1996; Lu et al. 2001; Takagi et al. 2001, 2002; Gottschalk et al. 2002).

Thus far, there are no experimental data on how the two integrin TM segments associate. NMR chemical shift data on the integrin β_3_ subunit TM-cytoplasmic domain fragment in dodecylphosphocholine micelles predict that the TM segment comprising residues Ile693 to Ile720 is largely α-helical (R. Li et al. 2002). Close apposition of the C-termini of the α_V_ and β_3_ extracellular domains in the crystal structure (Xiong et al. 2001) as well as specific interactions between α and β cytoplasmic tails (Vinogradova et al. 2002; Weljie et al. 2002) and cryoelectron microscopy of intact integrin α_IIb_β_3_ (Adair and Yeager 2002) suggest that the two TM segments are associated with each other as two interacting α helices, at least in the low-affinity state to which the crystal structure has been shown to correspond (Takagi et al. 2002). However, heterodimeric association between integrin α and β subunit fragments containing the TM and cytoplasmic domains has thus far not been detected in either detergent micelles (R. Li et al. 2001) or lipid bilayers, and association between the TM domains has never been demonstrated in intact cells. Since glycophorin A TM domains dimerize in lipid and detergent micelles (Lemmon et al. 1992) under conditions similar to those under which integrin TM domains fail to heterodimerize, it has been proposed that the interaction between the integrin TM domains is less stable (Gottschalk et al. 2002). Recently, R. Li et al. (2003) reported that both the integrin α and β subunits' TM helices have the potential to undergo homomeric rather than heteromeric interactions, and that stabilization of homooligomerization of integrin TM segments results in integrin activation. Li et al. hypothesize that the homomeric associations between TM segments provide a driving force for integrin activation. Experimental data on the association between integrin TM domains in intact cells are clearly required to decide between the many different models for how conformational signals are transmitted through the membrane in integrins.

Here we present extensive experimental evidence using cysteine mutagenesis and disulfide bond formation that integrin α and β TM segments associate with each other with a specific spatial orientation in the resting state. Mutations in the α subunit cytoplasmic tail known to universally activate integrins disrupt the heterodimeric TM domain interaction, but do not result in homomeric interaction. The effects of activating mutations are reversed by disulfide bond formation between α and β subunit TM domains. The results suggest that lateral separation of TM segments is responsible for the initial conversion to the high-affinity receptor.

## Results

### Structure of the TM Domain of Integrin α_IIb_β_3_ in the Resting State

#### Cysteine scanning of integrin TM domains

**Figure 1:**
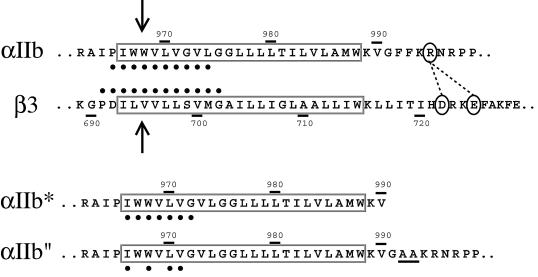
Sequences of the α_IIb_ and β_3_ TM Regions Segments predicted as TM by TMHMM version 2.0 (Krogh et al. 2001) are boxed. The more C-terminal KVGFF sequence in α_IIb_ and KLLITI sequence in β_3_ are additionally predicted to be in the membrane by Armulik et al. (1999). Charged residues involved in intersub-unit salt bridges (dotted lines) in the NMR cytoplasmic domain structure (Vinogradova et al. 2002) are marked with ovals. Residues used for cysteine scanning in this study are indicated by heavy dots. Arrows show the boundary between residues forming disulfide bonds constitutively and after oxidation. The se-quences of the α_IIb_* GFFKR truncation and α_IIb_" GFFKR/GAAKR mutants are also shown.

Inspection of the primary sequences of integrin subunits readily identifies putative TM segments of approximately 23 hydrophobic amino acids, as widely reported in the literature and as confirmed using a hidden Markov model approach (TMHMM version 2.0) (Krogh et al. 2001) (Figure 1). However, Armulik et al. (1999) experimentally determined the C-terminal boundary of both TM domains in microsomal membranes by introducing N-glycosylation sites at varying positions relative to the membrane, and found that the TM domains extend five or six residues more C-terminally and include a five-residue Lys-Val-Gly-Phe-Phe (KVGFF) sequence in α and a six-residue Lys-Leu-Leu-Ile-Thr-Ile (KLLITI) sequence in β (Figure 1).

**Figure 2:**
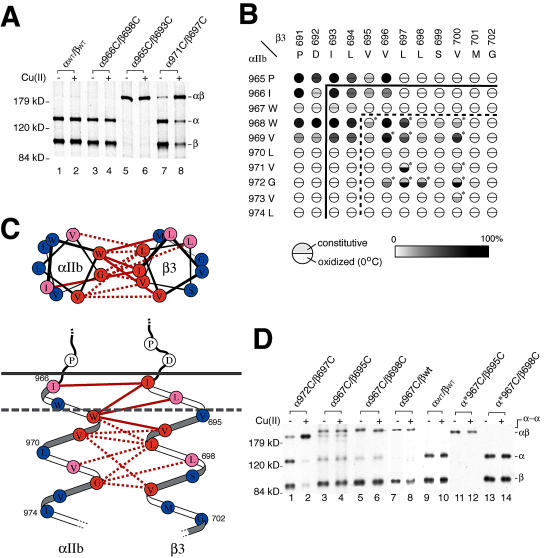
Formation of Intersubunit Disulfide Bonds in the TM Domain of Resting α_IIb_β_3_ (A) 293T cells were transiently transfected with the indicated integrin constructs and metabolically labeled, and were untreated (–) or oxidized with Cu-phenanthroline on ice for 10 min (+), and then lysates were immunoprecipitated with mouse mAb 10E5 against α_IIb_β_3_, followed by SDS-7.5% PAGE under nonreducing conditions and fluorography. Positions of molecular size markers are shown on the left. (B) Disulfide bond formation efficiency. For each residue pair, the radioactivity of the αβ heterodimer band divided by the total radioactivity (sum of α, β, and αβ bands) was used to calculate the disulfide bond formation efficiency and is depicted by a gray scale (white for 0% to black for 100% efficiency). The upper and lower halves of the circle indicate the efficiency before (constitutive) and after (oxidized) Cu-phenanthroline treatment at 0 °C, respectively. Residue pairs that form inducible disulfides (i.e., efficiency increases more than 10% after oxidation) are denoted by asterisks. Results are the mean of at least two independent experiments. Solid line shows the predicted TM boundary; dotted line indicates boundary between residues that form constitutive and inducible disulfide bonds. (C) Relative orientation of the α_IIb_ and β_3_ TM helices near their N-terminal ends. The TM domains are depicted schematically as α helices, and experimental results from cysteine scanning were used to deduce their relative orientation. The resultant schematic model is shown in both top and side views. Residue pairs that form disulfide bonds at greater than 50% efficiency are connected by solid (constitutive disulfides) or dotted (inducible disulfides) red lines. The gray dotted line represents the boundary between residues that form constitutive and inducible disulfide bonds. Residues are color coded based on the number of constitutive or inducible disulfide bonds formed at greater than 50% efficiency: multiple bonds (interacting residues, red), only one bond (peripheral residues, pink), and no bonds (outside residues, blue). (D) Homodimer formation by the W967C mutant of α_IIb_. Transfection, radiolabeling, and immunoprecipitation was performed as in (A). Full-length α_IIb_ with the W967C mutation (α-W967C) but not the truncated active mutant α_IIb_ with W697C (α*-W967C) produced a homodimer band (α–α) larger than the heterodimer band (α–β). The α972C/βL697C combination that produces efficient inducible heterodimer is shown as a standard (lanes 1 and 2).

In order to deduce the three-dimensional organization of the integrin TM domains, we utilized cysteine-scanning mutagenesis (Lee et al. 1995). Cysteine mutations were sequentially introduced at Pro965 to Leu974 of α_IIb_ and Pro691 to Gly702 of β_3_ (Figure 1) to give ten different α_IIb_ and 12 different β_3_ mutants, each containing a single cysteine residue. Mutant α_IIb_ and β_3_ chains were then cotransfected into 293T cells, biosynthetically labeled with [^35^S]-methionine and cysteine, and chased for 17 h with medium containing 500 μg/ml of cysteine and 100 μg/ml of methionine. Detergent cell extracts were immunoprecipitated with a monoclonal antibody (mAb) specific to the α_IIb_β_3_ complex and subjected to sodium dodecyl sulphate polyacrylamide gel elecrophoresis (SDS-PAGE). Because of the extensive chase, only mature, cell-surface α_IIb_β_3_ with complex N-linked glycan was isolated, which can readily be distinguished from the lower M_r_ α_IIb_ and β_3_ precursors with high mannose N-linked glycans (data not shown). When the two cysteines on the α and β subunits are spatially close and are oxidized during biosynthesis, they form a disulfide bridge that can be detected by the appearance of a covalently attached αβ heterodimer band in nonreducing SDS-PAGE with a concomitant decrease in the intensity of α and β monomer bands (e.g., Figure 2A, lane 5 compared to 1). Cysteines located near one another in the extracellular environment or in the membrane near the extracellular surface form disulfide bonds during the normal course of protein biosynthesis and processing. However, cysteines located more deeply in the membrane form disulfides much more efficiently when cells are treated with an oxidation catalyst such as Cu(II)-(*o*-phenanthroline)_3_ (Cu-phenanthroline) (e.g., Figure 2A, lane 8 compared to 7). Wild-type α_IIb_ and β_3_ subunits do not contain any cysteine residues in their TM domains and appear as 135- and 105-kDa bands, respectively, even after oxidation with Cu-phenanthroline (Figure 2A, lanes 1 and 2).

We tested all possible combinations between the ten α_IIb_ cysteine mutants (965C to 974C) and the 12 β_3_ cysteine mutants (691C to 702C), i.e., a total of 120 different cysteine pairs. Transient transfection in 293T cells and CHO cells gave similar results. The disulfide bonding efficiency of all of these pairs is graphically summarized in Figure 2B. All can be classified into three groups based on their ability to form disulfide-linked αβ heterodimers. The first group includes 17 pairs that constitutively formed disulfides with moderate (20%) to high (100%) efficiency and showed no increase in disulfide-bonded heterodimers upon Cu-phenanthroline treatment (Figure 2B, light to dark gray in both the upper and lower halves of individual circles). For example, cysteine pair α-P965C/β-I693C formed a disulfide-bonded dimer with an apparent molecular weight of 200 kDa at greater than 95% efficiency even without Cu-phenanthroline treatment (Figure 2A, lanes 5 and 6), suggesting that these two residues are in close proximity to each other near the exofacial membrane surface. The second group includes 13 pairs (Figure 2B, asterisk) that formed disulfides that increased in efficiency by 10% or more upon treatment with oxidant. For example, the efficiency of disulfide formation by the α-V971C/β-L697C pair was about 5% in the absence of oxidant (Figure 2A, lane 7) and about 70% after treatment of cells with Cu-phenanthroline at 0 °C for 10 min (Figure 2A, lane 8). The residues that formed disulfide bonds with increased efficiency after Cu-phenanthroline treatment are located deeper in the plasma membrane. The boundary between positions where disulfide bonds were constitutive and where they were increased by oxidants was between Trp967 and Trp968 in α_IIb_ and Leu694 and Val695 in β_3_ (dashed line in Figure 2B and solid arrows in Figure 1). The same results were obtained using oxidation with molecular iodine (I_2_), except that the efficiency of disulfide induction was slightly lower (data not shown). The third group, corresponding to the remaining 90 pairs, showed little or no intersubunit disulfide bond formation even after treatment with oxidant (Figure 2B, white in both upper and lower semicircles, and 2A, lanes 3 and 4).

#### Helical conformation of the TM domain and the interface between two interacting helices

The helical portions of the integrin TM domains are predicted to begin with residues α_IIb_-Ile966 and β_3_-Ile693 (Krogh et al. 2001), and the latter boundary is also suggested by NMR chemical shift data (R. Li et al. 2002) (solid lines in Figure 2B and the lower portion of 2C). A helical structure for the integrin α and β subunit TM domains was confirmed by formation of disulfide bonds with a helical periodicity in the entire portions of these segments scanned, corresponding to residues 966–974 in α_IIb_ and 693–702 in β_3_, i.e., approximately three α-helical turns in each (Figure 2B). Thus, α_IIb_ residue 965 constitutively formed disulfides while residue 967 did not, residues 968 and 969 formed constitutive and induced disulfides while 970 did not, and residues 971 and 972 formed induced disulfide bonds. A similar pattern was seen in β_3_, with the minima in disulfide formation efficiency occurring at residues 695, 698/699, and 701/702. This periodicity and the disulfide bonding pattern shown below demonstrate a helical structure.

To determine the approximate orientation between the α_IIb_ and β_3_ TM helices, the data on disulfide formation were mapped onto a helical wheel representation (Figure 2C, upper portion) and an orthogonal view with the axes of the helices in the plane of the page (Figure 2C, lower portion). Both the overall disulfide-bond-forming efficiency of individual residues and the pattern of disulfide bond formation are consistent with a unique orientation between the two helices in terms of both the faces of the two helices that are apposed (Figure 2C, upper portion) and the relation between the two helices in their axial directions (Figure 2C, lower portion). Furthermore, the axial relationship deduced from this pattern is identical to that obtained by assuming that the boundary between residues that form constitutive and inducible disulfide bonds should be at the same depth in the membrane for both helices (gray dashed line in the lower portion of Figure 2C).

#### Single and double cysteine mutants are in the low-affinity state

On both CHO-K1 (Kashiwagi et al. 1999) and 293T transfectants, α_IIb_β_3_ has low affinity for soluble ligand. As shown below, none of the double mutants that formed disulfide bonds bound ligand spontaneously. Furthermore, none of the ten β_3_ or 12 α_IIb_ single-cysteine mutants studied here showed elevated ligand binding activity (data not shown). Consistent with this, studies on dimerization of the glycophorin A TM domains have shown that cysteine substitutions are on average less disrupting than substitutions with any other hydrophobic residue (Lemmon et al. 1992). We conclude that the α/β TM domain association depicted in Figure 2C is that of the resting (low-affinity) integrin conformation.

#### Formation of tetrameric receptors with the α-W967C mutant

When cysteine mutant α-W967C was used, a high-molecular-weight species that migrated more slowly than the heterodimer appeared in nonreducing gel electrophoresis, accompanied by a decrease in the intensity of the α_IIb_ band but not of the β_3_ band (Figure 2D, lanes 3 and 5 compared to lane 9). Treatment with Cu-phenanthroline did not further increase the intensity of the new band (Figure 2D, lanes 4 and 6). In reducing SDS-PAGE, the high-molecular-weight band disappeared and was converted into monomeric α_IIb_ (data not shown). Furthermore, the same high-molecular-weight band was observed when α-W967C was cotransfected with any of the β_3_ cysteine mutants (β-V695C and β-L698C are shown as examples in lanes 3–6 in Figure 2D) as well as with wild-type β_3_ (Figure 2D, lanes 7 and 8) at a similar efficiency of about 80%, confirming that it was an α–α dimer. Furthermore, α–α cross-linking did not affect α–β association, because a stoichiometric amount of β_3_ was immunoprecipitated (Figure 2D), and the amount of immunoprecipitation by the αβ complex-specific mAb 10E5 was unaffected. Therefore, disulfide linkage through α-W967C results in the formation of a tetramer in which two α_IIb_β_3_ heterodimers are covalently linked through a Cys967–Cys967 disulfide bond to form a (α_IIb_β_3_)_2_ tetramer. Notably, among the ten α and 12 β cysteine mutants used in this study, only α-W967C formed a homodimeric disulfide bond. This is consistent with the model of α–β TM domain association deduced here (Figure 2C), because residue Trp967 faces outward, away from the interface with β_3_. Furthermore, constitutive formation of the Cys967–Cys967 disulfide bond is consistent with the location of Trp967 in the exofacial portion of the α_IIb_ TM α helix, where disulfide bonds form constitutively (Figure 2C, lower portion).

### Disulfide-bonded Receptor Can Be Activated from Outside by Mn^2+^ and mAb

**Figure 3:**
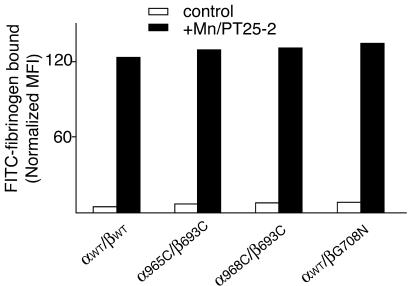
Disulfide-bonded Receptors Can Be Activated from Outside the Cell Transiently transfected 293T cells expressing wild-type (αwt/βwt) or mutant α_IIb_β_3_ heterodimers that form constitutive disulfide bonds (α965C/β693C and α968C/β693C) or are reported elsewhere to be activated (αwt/βG708N) (R. Li et al. 2003) were incubated with FITC-fibrinogen in a physiological buffer (control, white bars) or in the presence of 1 mM Mn^2+^ and the activating mAb PT25–2 (+Mn/PT25–2, black bars). Binding of FITC-fibrinogen was determined by flow cytometry as the mean fluorescence intensity and normalized by dividing by the mean fluorescence intensity with Cy3-labeled anti-β_3_ mAb AP3 and multiplying by 100.

Previous work has shown that substitution of integrin α_L_ and β_2_ subunit cytoplasmic domains for α helices that form a noncovalently associated α-helical coiled-coil heterodimer stabilizes the low-affinity state and is dominant over intracellular signaling pathways that activate integrins; nonetheless, such constructs can be activated from outside the cell by activating mAb or Mn^2+^ (Lu et al. 2001). Consistent with this finding, activation of integrin α_L_β_2_ with Mn^2+^ does not result in separation of the native cytoplasmic domains tagged with fluorescent proteins (Kim et al. 2003). To test whether the covalent disulfide linkage of the integrin α and β subunit TM domains prevents α_IIb_β_3_ from being activated from the outside by mAb and Mn^2+^, soluble ligand binding was measured. The 293T cell transfectants expressing wild-type α_IIb_β_3_ did not bind soluble fibrinogen in a physiological buffer containing Ca^2+^ and Mg^2+^, but high-affinity binding was observed in the presence of Mn^2+^ and the activating mAb PT25–2 (Figure 3). Two cysteine mutants with approximately 100% constitutive disulfide bond formation, α-P965C/β-I693C and α-W968C/β-I693C (see Figure 2A and 2B), were tested in parallel. Fibrinogen binding by these disulfide-bonded mutants was activated by Mn^2+^ and PT25–2 mAb indistinguishably from wild type (Figure 3). Similar results were obtained after Cu-phenanthroline–induced disulfide bond formation in mutants with cysteine substitutions deeper in the membrane, α-V971C/β-L697C and α-G972C/β-L697C (data not shown). These data demonstrate that even a covalent clasp at the TM domain cannot maintain integrins in the inactive state if they are activated from outside the cell by mAb and Mn^2+^.

### Separation of TM Helices Upon Integrin Activation from Inside the Cell

**Figure 4:**
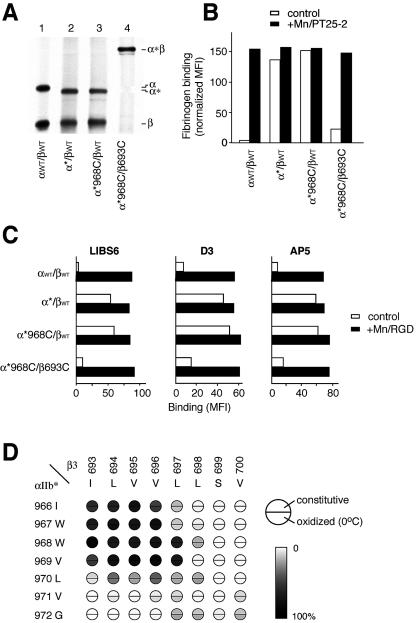
Formation of Intersubunit Disulfide Bonds in the TM Domain of α_IIb_*β_3_ and Effect on Ligand Binding and LIBS Epitopes (A) Immunoprecipitation. Immunoprecipitation of [^35^S]-labeled receptors and nonreducing SDS-PAGE and fluorography was as described in Figure 2. (B) FITC-fibrinogen binding. Binding was determined by immunofluorescence as described in Figure 3. (C) LIBS exposure. Three different anti-LIBS mAbs (LIBS6, D3, and AP5) were used to probe the conformational state. mAb binding is expressed as the mean fluorescence intensity in the absence (control, open bars) or presence (+Mn/RGD, black bars) of Mn^2+^ and RGD peptide. (D) Disulfide bond formation efficiency. Disulfide bond formation in α_IIb_*β_3_ heterodimers with the indicated residues mutated to cysteine was determined as described in Figure 2B.

Is the specific TM helix association defined here disrupted in response to activation from inside the cell? We mimicked physiological inside-out integrin activation by using α_IIb_β_3_ containing a truncation before the Gly-Phe-Phe-Lys-Arg (GFFKR) motif in the α_IIb_ subunit (O'Toole et al. 1994), or a Gly-Ala-Ala-Lys-Arg (GAAKR) sequence in place of the GFFKR sequence (Lu and Springer 1997; Kim et al. 2003) (see Figure 1). When cotransfected with the wild-type β_3_ subunit, α_IIb_ truncated at Gly991 (denoted α*) formed a heterodimer on the cell membrane and appeared as an approximately 130-kDa band, slightly smaller than wild-type α_IIb_ in nonreducing SDS-PAGE (Figure 4A, lane 2). Transfectants expressing the mutant α*/β receptor bound soluble fibrinogen in the absence of any activation, confirming the activating effect of C-terminal truncation (Figure 4B). Furthermore, the α*/β heterodimers constitutively expressed three independent activation-dependent epitopes called ligand-induced binding sites (LIBSs) in the absence of ligand (Figure 4C, α*/βwt), demonstrating conversion of the extra-cellular domain to the extended conformation (Takagi et al. 2002).

Using this active α* mutant, cysteine scanning was performed. As shown in Figure 4D, the results were very different from those obtained with full-length α_IIb_β_3_ in two important respects. (1) No periodicity in disulfide formation was observed (Figure 4D). The only pattern was that the more N-terminal exofacial residues preferentially bonded to more exofacial residues in the other subunit, whereas more buried residues preferentially bonded to more buried residues in the other subunit. The lack of periodicity is highly unlikely to result from a loss of helical secondary structure in such a large portion of the TM domains (S. C. Li and Deber 1993). Furthermore, even in a dodecylphosphocholine detergent environment and in the absence of association with α_IIb_, this portion of the β_3_ TM domain retains an α-helical structure as shown by NMR experiments (R. Li et al. 2002). Therefore, the loss of periodicity in disulfide formation suggests that there is no longer a preferred orientation between the α and β subunit TM helices. (2) Oxidant-induced disulfide bond formation at 0 °C was not observed (Figure 4D). As shown below, this is because in the absence of constitutive disulfide bond formation, the TM domains of the α*/β heterodimers are not, or are only transiently, associated with one another in the membrane.

**Figure 5:**
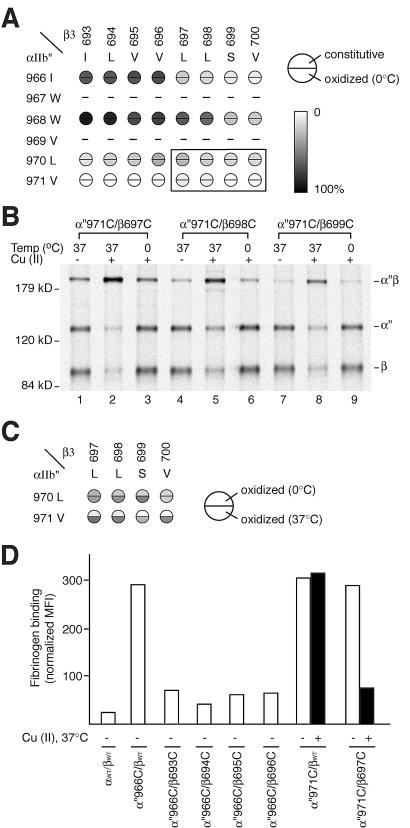
Formation of Intersubunit TM Disulfide Bonds in GFFKR/GAAKR Mutant α_IIb_′′β_3_ Receptors and Effect on Ligand Binding (A) Disulfide bond formation efficiency in α_IIb_"β_3_. Disulfide bond formation in α_IIb_"β_3_ heterodimers with the indicated residues mutated to cysteine was determined as in Figure 2B. Boxed residue pairs were also subjected to Cu-phenanthroline oxidation at 37 °C in (B and C). (B) Radiolabeled 293T cells expressing the indicated mutant integrins were treated with Cu-phenanthroline at 0 °C or 37 °C, followed by immunoprecipitation with anti-α_IIb_β_3_, SDS-PAGE, and fluorography to probe disulfide bond formation. (C) Efficiency of intramembranous disulfide bond formation in the context of the α_IIb_"β_3_ mutant receptor was assessed after Cu-phenanthroline oxidation at 0 °C or 37 °C and expressed as in Figure 2B. (D) FITC-fibrinogen binding. Binding was determined before (–) and after (+) Cu-phenanthroline oxidation at 37 °C and expressed as in Figure 3.

We thought it important to confirm these results with an activated integrin that was not truncated and therefore used α_IIb_ with the GFFKR sequence mutated to GAAKR, designated α_IIb_". A smaller number of cysteine-scanning substitutions were introduced into α_IIb_", and tested together with the β_3_ cysteine mutants (Figure 5A). The same two major trends were found as with α_IIb_*/β_3_. (1) Just as in α_IIb_*/β_3_, in α_IIb_"/β_3_, the helical periodicity of disulfide bonding was lost, as evidenced by the results with the β_3_ scanning mutants β-I693 to β-V700 (Figure 5A). (2) As found with α_IIb_*/β_3_ and not with α_IIb_/β_3_, none of the α_IIb_"/β_3_ mutants showed increased disulfide bond formation when treated with Cu-phenanthroline at 0 °C (Figure 5A and 5B).

It is significant that a number of α*/β and α"/β cysteine-scanning mutants could form disulfide-bonded heterodimers during biosynthesis, but in contrast to α/β, none showed increased disulfide formation after oxidation at 0 °C. During biosynthesis at 37 °C, the membrane is fluid. Disulfide bond formation is catalyzed in the endoplasmic reticulum by disulfide isomerases, and because the redox balance is oxidizing in the endoplasmic reticulum, disulfide bond formation can covalently trap protein complexes that form only transiently. Therefore, a complex that would not be stable energetically by noncovalent interactions alone may nonetheless be stabilized by a covalent disulfide bond. This may particularly be the case for interactions between integrin TM domains, because the noncovalent association between the α and β subunits in the headpiece in the extracellular domain increases the probability of collision between the α and β subunit TM domains. If disulfide formation is the result of a stable noncovalent interaction between TM domains, it should occur at 0 °C when membranes are in a gel phase and proteins do not diffuse, as well as at 37 °C when membranes are liquid-crystalline and proteins diffuse. On the other hand, if disulfide formation is the result of transient interactions that are energetically unfavored, it should occur at 37 °C but not at 0 °C.

**Figure 6:**
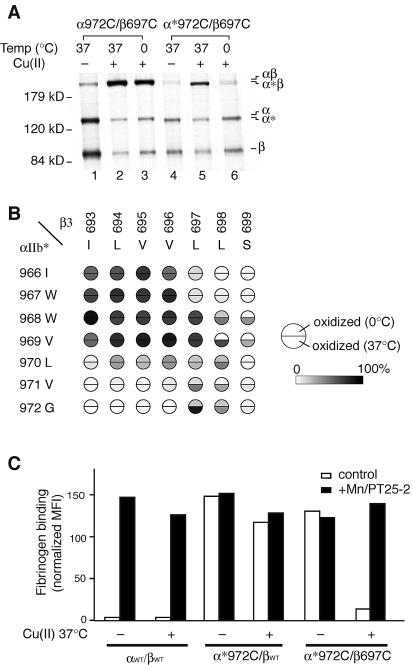
Formation of an Intersubunit Disulfide Bridge within the Membrane Reverses the Active Phenotype of the α_IIb_*β_3_ Receptor (A) Radiolabeled 293T cells expressing the indicated mutant integrins were treated with Cu-phenanthroline at 0 °C or 37 °C, followed by immunoprecipitation with anti-α_IIb_β_3_, SDS-PAGE, and fluorography to probe disulfide bond formation. (B) Efficiency of intramembranous disulfide bond formation in the context of the truncated α_IIb_*β_3_ receptor was assessed after Cu-phenanthroline oxidation at 0 °C or 37 °C and expressed as in Figure 2B. (C) Ligand binding by wild-type or mutant α_IIb_*β_3_ expressed on 293T cells was determined before (–) and after (+) Cu-phenanthroline oxidation at 37 °C and expressed as in Figure 3.

To confirm the hypothesis that in α*/β and α"/β transient collision between TM helices can result in disulfide formation, Cu-phenanthroline oxidation was performed both at 0 °C and 37 °C. As described above, the α-G972C/β-L697C pair in the context of the wild-type receptor shows greatly increased disulfide bond formation upon oxidation by Cu-phenanthroline at 0 °C (Figure 6A, lane 3 compared to 1). In contrast, the same residue pair in the context of the truncated active mutant, α*-G972C/β-L697C, did not show increased disulfide bond formation after oxidation at 0 °C (Figure 6A, lane 6 compared to 4). When oxidation was performed at 37 °C, however, this intramembranous disulfide bond formed in the context of the truncated α*/β mutant (Figure 6A, lane 5). This strongly supports the hypothesis that association of the TM segments in the α*/β receptor is not energetically favored—and is thus present only in an undetectably small subpopulation of molecules at any one moment—but is a kinetically accessible state in a fluid membrane at 37 °C that can be trapped by disulfide formation. Increased disulfide bond formation by α*/β mutants by oxidation at 37 °C was not due to increased catalysis by Cu-phenanthroline or other nonspecific factors, because in full-length α/β, disulfide linkage induced by Cu-phenanthroline was the same at 37 °C (data not shown) as at 0 °C (see Figure 2B). Oxidation-induced cross-linking at both 0 °C and 37 °C was extended to all other cysteine pairs in the context of the α*/β mutant (Figure 6B). Nine of them showed significant enhancement in cross-linking at 37 °C compared to 0 °C (Figure 6B), whereas none of the same pairs in full-length αβ showed enhanced cross-linking at 37 °C compared to 0 °C (Figure 6A, lanes 1–3, and data not shown).

The above results were confirmed with the full-length α"/β receptor containing the Phe-Phe/Ala-Ala substitution (see Figure 5B and 5C). Thus, Cu-phenanthroline did not increase disulfide bond formation between buried residues at 0 °C (Figure 5A and 5B), but it markedly increased disulfide bonding at 37 °C (Figure 5B and 5C). Taken together, the above results demonstrate that (1) integrin α and β subunit TM helices separate from one another upon activation from inside the cell, (2) transient association between TM helices in activated receptors can be trapped either by disulfide bond formation during biosynthesis or by Cu-phenanthroline oxidation at 37 °C, and (3) in activated receptors the specific pattern of association between the TM helices seen in the resting state is not present.

A further important finding was that none of the cysteine mutants, including the W967C mutant of α_IIb_ which mediated α–α homodimerization in the wild-type receptor, underwent α–α homodimerization in the context of the activated α*/β receptor (see Figure 2D, lanes 11–14). In contrast, the same cysteine combinations formed α–α homodimers in the context of the full-length αβ receptor without the activating mutation (Figure 2D, lanes 3–8). This result is inconsistent with the notion that homooligomerization of TM domains occurs concomitantly with separation of the α and β subunit TM domains and represents the major mechanism for inside-out activation of integrins.

### TM Helix Separation Is Responsible for Activation of Integrins from within the Cell

As described above, integrins with disulfide-linked TM domains can be activated from the outside by Mn^2+^ and mAb; however, we now demonstrate that such a linkage prevents activation from the inside. We first examined the activation state of receptors with activating α* or α" mutations that constitutively form disulfide bonds during biosynthesis. When α*-W968C was coexpressed with β-I693C, nearly 100% formation of the intersubunit disulfide linkage was observed (see Figure 4A, lane 4). This cross-linked receptor showed low basal ligand binding activity, like the wild-type receptor (Figure 4B). By contrast, α*-W968C/βwt (Figure 4B) and α*/β-I693C (data not shown), which did not form cross-links, were basally active (Figure 4B). The disulfide cross-link had no deleterious effect on ligand binding itself because, as already mentioned above, α*-W968C/β-I693C bound ligand upon activation by Mn^2+^ and activating mAb (Figure 4B). Furthermore, in α*-W968C/β-I693C but not in α*-W968C/βwt or α*/β-I693C (data not shown), the exposure of activation-dependent epitopes was reduced to the level of the wild-type receptor (Figure 4C). This result suggests that the shift from the bent to the extended conformation induced by the activating α* mutation was reversed by the TM disulfide bond. The same reversal of basal ligand binding, but not Mn^2+^/PT25–2-activated ligand binding, was found for all constitutively disulfide-bonded α*/β pairs that we examined. These included α*-I966C/β-I693C, α*-I966C/β-L694C, α*-I966C/β-V965C, α*-W968C/β-L694C, α*-W968C/β-V695C, α*-W968C/β-V696C, and α*-W968C/β-L697C (data not shown). The same reversal of basal ligand binding was also found for all constitutively disulfide-bonded α"/β pairs examined, including α"-I966C paired with either β-I693C, β-L694C, β-V695C, or β-V696C (see Figure 5D). Therefore, a wide range of distinct intersubunit cross-links in the outer leaflet of the membrane reverse, and are hence dominant over, activating mutations in the α subunit at the boundary between the membrane and the cytoplasm.

Could a receptor that was already present at the cell surface and active in ligand binding be converted to an inactive receptor by introduction of a disulfide bond between the α and β subunit TM domains? We were able to answer this question by using the more buried pairs of cysteine residues that formed disulfide bonds upon oxidation catalyzed by Cu-phenanthroline. In α*/β we studied the α*-G972C/β-L697C pair, which shows greatly enhanced disulfide bond formation after treatment with Cu-phenanthroline at 37 °C (see Figure 6A and 6B). Under basal conditions, the α*-G972/β-L697C mutant actively binds fibrinogen (Figure 6C). However, after Cu-phenanthroline treatment at 37 °C, basal ligand binding was almost completely lost, but ligand binding activatable by Mn^2+^/PT25–2 mAb was still present (Figure 6C). Cu-phenanthroline treatment at 37 °C was not toxic for basal ligand binding, because the same treatment did not reverse basal ligand binding by α*-G972C/βwt (Figure 6C) or α*/βwt (data not shown).

These results were extended to the α"/β mutant using a different pair of cysteines in the α"-V971C/β-L697C mutant that shows Cu-phenanthroline-induced disulfide bond formation at 37 °C (see Figure 5B and 5C). Cu-phenanthroline treatment at 37 °C almost completely reversed the elevated basal ligand binding by α"-V971C/β-L697C, but had no effect on α"-V971C/βwt (Figure 5D). We conclude (1) that at 37 °C, the α*-G972C/β-L697C and α"-V971C/β-L697C heterodimers are predominantly in an active conformation with separated TM domains but equilibrate with a conformation in which the TM domains are transiently associated, and (2) that when association of the TM domains is trapped by disulfide bond formation, the ligand binding site in the extracellular domain returns to the low-affinity state.

## Discussion

We have obtained for the first time structural information regarding the helix–helix interface between integrin α and β subunit TM domains in the membrane bilayer, and demonstrate that dissociation at this interface occurs upon changes at the cytoplasmic face of the plasma membrane bilayer that activate integrins. Extensive mutagenic cysteine cross-linking experiments revealed the presence of a specific α/β TM helix contact in a resting integrin heterodimer, which is lost upon receptor activation from inside the cell. The data establish the approximate orientation between the integrin TM α helices in the outer leaflet of the membrane bilayer in the resting, low-affinity integrin conformation (see Figure 2C).

The mode of association experimentally determined here may be compared to that suggested by computational models (Gottschalk et al. 2002). For comparisons, we used our cross-linking data to construct a model by selecting an alignment to the glycophorin A TM homodimer NMR structure (Mac-Kenzie et al. 1997) that minimized the distances between residues with more than 80% cross-linking efficiency (see Materials and Methods). The overall orientation in our model is not too dissimilar from that of a model for the resting conformation of the α_IIb_β_3_ TM domains (Gottschalk et al. 2002), but our model fits the data better, with a root mean square distance for three Cβ–Cβ and two Cβ–Gly Cα atom distances of 4.8 Å, compared to 8.9 Å for the computational model. Furthermore, our cross-linking data on the activated receptor are completely incompatible with a model for the activated TM domain interface (Gottschalk et al. 2002) because the cross-linked regions in α_IIb_ and β_3_ are close together in this model, yet a specific pattern of cross-linking predicted by the model was not observed.

Integrin TM domain homodimerization and heterodimerization has been assayed using a qualitative assay of induction of β-galactosidase in Escherichia coli (Schneider and Engelman 2003). However, the chimeras that were assayed contain truncated integrin TM domains with only 17 residues of the α and β subunit TM domains, lack the GFFKR motif demonstrated here to be required for physiologic TM domain association, and insert as type II rather than type I membrane proteins. These assays were designed to test the hypothesis that the Gly-Val-Met-Ala-Gly (GVMAG) homodimerization motif in glycophorin is equivalent to G972/VLGG in α_IIb_ and S699/VMGA in β_3_ (Schneider and Engelman 2003). However, the use of the glycophorin template (MacKenzie et al. 1997) to fit our experimental data demonstrates that the GVMAG dimerization interface is more equivalent to α_IIb_-W968/VLVG and β_3_-V696/LLSV (see Materials and Methods).

**Figure 7:**
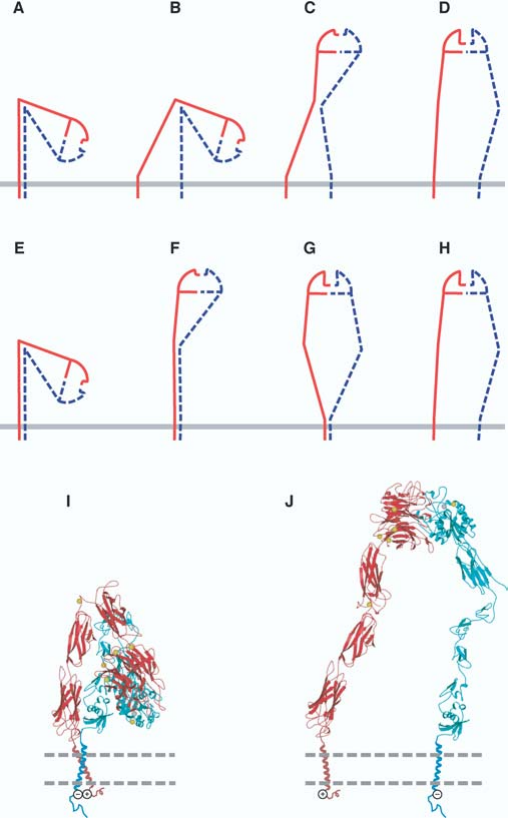
Model for Integrin Activation The α and β subunits are red and blue, respectively. The membrane is shown as a solid gray line in (A–H) and as two dashed lines in (I and J). (A–H) Cartoon models. The ligand-binding α subunit β-propeller and β subunit I-like domains are symbolized as a semicircle with a shallow (low-affinity) or deep (high-affinity) ligand binding site. The headpiece additionally contains the α subunit thigh domain (red straight line) and the β subunit hybrid domain (blue straight line); the swing out of the latter is linked to ligand binding affinity. (A–D) Activation from within the cell initiated by TM domain separation. (E–H) Activation from outside the cell initiated by integrin extension, followed by ligand binding and finally TM domain separation. (I and J) Ribbon models. (I) Bent, low-affinity conformation corresponding to (A and E). (J) Extended, high-affinity conformation with the open headpiece corresponding to (D and H). Models are based on the TM domain association results described here, and negative stain electron microscopy (Takagi et al. 2002, 2003), crystallography (Xiong et al. 2001), NMR (Beglova et al. 2002; Vinogradova et al. 2002), and fluorescent resonance energy transfer (Kim et al. 2003). The TM and cytoplasmic domains are schematic, and show the proposed salt bridge (– and +).

Our cysteine cross-linking data not only define the nature of the interface between the α and β subunit TM domains within integrin heterodimers but also provide information about the spatial relationship between neighboring integrin heterodimers on the cell surface. The formation of a cross-link between the α_IIb_ subunits of two neighboring integrin molecules by the α_IIb_-W967C mutant demonstrates the lateral accessibility of this site in the resting state. Consistent with this finding, our data demonstrate that the α_IIb_-W967 residue points away from the TM interface with the β_3_ subunit (see Figure 2C). It further should be noted that in the bent, low-affinity integrin conformation present on the cell surface (Takagi et al. 2002), the headpiece is folded such that the juxtamembrane portion of the α_IIb_ subunit, including Trp967, is exposed, whereas the juxtamembrane segment of β is occluded (Figure 7). This is consistent with the absence of homodimer cross-linking through β_3_.

It is most interesting that we observed no homodimerization with constitutively active mutant receptors. The α and β residues mutated to cysteine in active receptors spanned two and three α-helical turns in the α_IIb_ and β_3_ TM domains, respectively. The same mutations in resting receptors robustly disclosed heterodimeric interactions. Therefore, if homodimeric interactions between the TM domains were present, they should have been detected. Why were homodimer interactions observed in the resting state, albeit only through cross-linking of one residue, and not in the active state? A full answer to this question would require more knowledge about the dynamics of integrins on cell surfaces; however, based on observations on the heterogeneity of integrin structure from quantitative negative stain electron microscopy of soluble integrins (Takagi et al. 2002), a preliminary answer can be proposed. These studies reveal that the integrin adopts a single homogenous, bent conformation in the resting state. By contrast, in the extended conformation, there are two discrete angles between the β subunit I-like and hybrid domains. Furthermore, the region between the β subunit hybrid domain and the TM domain, which contains four I-EGF domains and the β-tail domain, is quite flexible. Therefore, motion of the headpiece may sweep out a large area and prevent neighboring integrins from coming close. Moreover, motions of the membrane proximal α subunit calf-2 domain relative to the α TM domain and of the β-tail domain relative to the β TM domain would also be much greater after TM domain and tailpiece separation, and would also hinder the close approach of other TM domains.

What about observations that integrin fragments consisting of the TM and cytoplasmic domains form dimers (α_IIb_) and trimers (β_3_) in detergent micelles (R. Li et al. 2001)? We think that these findings should be interpreted with caution. It is important to point out that the physiological, heterodimeric interaction between the α_IIb_ and β_3_ TM domains cannot be reconstituted in the same detergents, i.e., in sodium dodecyl sulfate or dodecylphosphocholine (R. Li et al. 2001). There are many important differences between dodecyl detergent micelles and lipid bilayers, including a shorter hydrocarbon chain (12 versus 16 or 18), one (as opposed to two) fatty acyl chains per headgroup, and a spherical (as opposed to a bilayer) shape. The same characteristics that prevent physiological heterodimeric integrin TM interactions in dodecyl detergent micelles may conspire to cause nonphysiologic homomeric interactions. A β_3_-G708N mutation increases trimerization in detergent by more than 10-fold and is also reported to activate ligand binding in transfectants (R. Li et al. 2003); however, β_3_ trimerization in membrane bilayers or intact cells has yet to be demonstrated. In 293T transfectants the β_3_-G708N mutation fails to detectably activate soluble ligand binding by α_IIb_β_3_ (see Figure 3). We could confirm that the G708N mutation in CHO cells increased ligand binding, but to a level only 17% of that of the maximally activated receptor, whereas the G708L mutation is maximally activating (data not shown). Gly708 is in the TM heterodimer interface defined here, and we have additional unpublished data suggesting that the weak activating effect of the G708N mutation is a consequence of the disruption of this interface. The lack of homomeric disulfide cross-linking of integrin α and β subunit TM domains found here with activated α_IIb_β_3_ in intact cells strongly suggests that integrin activation from inside the cell is not sufficient to drive homomeric interactions. Studies with fluorescent resonance energy transfer probes attached to integrin cytoplasmic domains also fail to find homomeric interactions when integrins are activated from within the cell or bind to monomeric ligand outside the cell (Kim et al. 2003; M. Kim, C. Carman, and T. Springer, unpublished data). However, we should point out that binding to multimeric ligands induces integrin clustering (Buensuceso et al. 2003) and that we have not examined homomeric interactions under these conditions. In conclusion, our results suggest that lateral separation of the TM segments of the α and β chains leads to affinity upregulation within a single receptor molecule without facilitating α–α or β–β interactions. Therefore, if the tendency of integrin TM domains to undergo homomeric interactions in detergent micelles also holds for lipid bilayers, it may strengthen adhesion and contribute to outside-in signaling after the initial engagement of multimeric physiological ligands.

Our results show that the α_IIb_ and β_3_ TM domains are associated in a specific manner in the outer leaflet of the membrane bilayer in the resting state and are unassociated in the active state. Upon activation, association between the α and β subunits is also broken at the interface between the TM and cytoplasmic domains (Hughes et al. 1996; Vinogradova et al. 2002), and furthermore, the cytoplasmic domains also separate (Kim et al. 2003). The simplest explanation for separation at all three of these locations is separation of the TM domains in the plane of the membrane. Perhaps a counterargument could be made that a hinge-like motion of the TM domains relative to one another about a pivot point near the middle of the bilayer would also give rise to separation at each of these three positions. We point out that only one specific TM hinge model has been proposed, that it does not give rise to separation in the TM regions scanned in this study (Gottschalk et al. 2002), that our data rule it out, and that much more extreme hinging is unprecedented and is unlikely, because the size of the TM interface would be markedly decreased and hence less likely to stabilize association.

Bidirectional signal transmission by integrins across the plasma membrane is not necessarily symmetric (Figure 7A–7D compared to 7E–7H). We show that separation of the TM domains is sufficient to prime the extracellular domain for ligand binding and exposes activation epitopes that report the switchblade-like extension of the extracellular domain (Figure 7A–7D). Furthermore, prevention or reversal of TM domain separation abolishes priming and extension signaled from the inside. The same is not true in the opposite direction (Figure 7E–7H); thus, addition of Mn^2+^ and an activating mAb to the extracellular environment could prime ligand binding in the absence of TM domain separation. The implication is that with the wild-type receptor, under conditions in which high concentrations of ligand drive the equilibrium toward ligand binding, ligand could bind in the absence of TM domain separation (Figure 7G) and subsequently drive TM domain separation (Figure 7H). Similarly, when separation of fluorescent resonance energy transfer tags fused to the C-termini of the cytoplasmic domains of integrin α_L_β_2_ is measured, priming from inside the cell results in TM domain separation (Figure 7B–7D), priming from outside the cell by Mn^2+^ does not result in separation (Figure 7F and 7G), and priming with Mn^2+^ combined with binding to ligand results in separation (Figure 7H) (Kim et al. 2003). Therefore, in Mn^2+^, integrins on the cell surface appear to adopt an intermediate conformation, with the headpiece extended and the TM domains associated (Figure 7F and 7G). The above results are consistent with the existence of multiple conformational states visualized for integrin extra-cellular domains by electron microscopy, and linked equilibria relating these states (Takagi et al. 2002). Furthermore, extended conformations with both closed and open headpieces are present in Mn^2+^ (Figure 7F and 7G), whereas only the extended conformation with the open headpiece is present in high concentration of ligand (Figure 7H) (Takagi et al. 2002, 2003).

How does TM domain separation trigger integrin extension? In the bent α_V_β_3_ crystal structure (Xiong et al. 2001), the last residue visualized in β_3_ is Gly690, immediately before the first TM domain residue mutated to cysteine here. In the α subunit, only four to six residues intervene between the last crystal structure residue and the first residue mutated to cysteine. This very tight linkage between the C-terminal extracellular domains and the TM domains (Figure 7I) implies that separation of the α and β TM domains would also lead to separation of the membrane proximal α calf-2 and β-tail domains in the integrin tailpiece. In turn, this separation in the tailpiece would destabilize the extensive interface with the headpiece and lead to switchblade opening (Figure 7J) (Takagi et al. 2002).

Separation of TM domains in the plane of the membrane is a novel mechanism for activation of a cell surface receptor. One of the best-known mechanisms for receptor activation, exemplified by receptor tyrosine kinases (Schlessinger 2000), works in almost the opposite manner, in which distinct or identical receptor subunits are brought together in a specific orientation in the plane of the membrane by ligand binding. In the neu (ErbB-2) member of the epidermal growth factor receptor family, enforced dimerization along a series of α-helical TM dimer interfaces gives rise to periodicity in activation, such that dimerization only in certain orientations is activating (Burke and Stern 1998; Bell et al. 2000). In our study, the α*β and α"β receptors with activating mutations were captured with disulfide bonds in many different rotational orientations between the α and β subunit TM α helices. Similarly, disulfide bonding between cysteines located at different depths in the membrane would be expected to give rise to some piston-like motion of one helix relative to the other. It is notable that none of the enforced orientations between disulfide-bonded α and β integrin TM domains were activating. These results argue against hinging, rotation, or piston models in which a relative change in orientation between the two TM domains is activating, and are in agreement with the model that separation of the α and β subunit TM domains in the plane of the membrane is the activation mechanism.

Integrins in the extended conformation have their ligand binding site far above the plasma membrane, as appropriate for binding to ligands in the extracellular matrix and on opposing cell surfaces. However, transmission of conformational information over such distances is inefficient, because it is attenuated by interdomain flexibility. Integrins solve the problem of long distance communication by equilibrating between an extended conformation and a bent conformation, and by altering the equilibrium between these conformations by the novel mechanism of separation of the α and β subunit TM domains.

## Materials and Methods

### 

#### Plasmid construction and transient transfection

Plasmids coding for full-length human α_IIb_ and β_3_ were subcloned into pEF/V5-HisA and pcDNA3.1/Myc-His(+), respectively, as described by Takagi et al. (2002). To mimic inside-out signaling, α_IIb_ cytoplasmic domain mutant receptors were made by introducing a stop codon at residue Gly991 to obtain α_IIb_1–990 (denoted α*), or by mutating G991/FFKR to GAAKR (denoted α"). Single amino acid substitutions to cysteine were made in α_IIb_, α_IIb_*, α_IIb_", and β_3_ in the positions indicated in the text. All mutants were made using site-directed mutagenesis with the QuikChange kit (Stratagene, La Jolla, California, United States), and DNA sequences were confirmed before transfection of 293T cells using calcium phosphate precipitates, or CHO-K1 cells using Fugene transfection kit (Roche Diagnostics, Indianapolis, Indiana, United States).

#### Cross-linking and immunoprecipitation

Twenty-four hours after transfection, 293T cells were metabolically labeled with [^35^S]cysteine/methionine for 1.5 h before adding chase medium containing 500 μg/ml of cysteine and 100 μg/ml of methionine, and cells were cultured 17 h overnight (Lu et al. 2001). Then cells were detached and suspended in Tris-buffered saline (TBS) containing 1 mM Ca^2+^/1 mM Mg^2+^ (10^6^ cells in 100 μl). After chilling on ice for 5 min, 20 μM CuSO4/100 μM *o*-phenanthroline was added by 10-fold dilution from stock solutions, and cells were incubated on ice for another 10 min. Oxidation at 37 °C was similar, except cells were suspended at room temperature and after Cu-phenanthroline addition were incubated at 37 °C for 10 min. Oxidation was stopped by adding an equal volume of TBS containing Ca^2+^/Mg^2+^ and 5 mM N-ethyl maleimide. Cells were centrifuged and resuspended in 100 μl of TBS containing 1 mM Mg^2+^, 1 mM Ca^2+^, and 5 mM N-ethylmaleimide, and lysed by addition of an equal volume of 2% Triton X-100 and 0.1% NP-40 in the same buffer for 10 min on ice. Cell lysate was immunoprecipitated with 10E5 (anti-α_IIb_β_3_-complex-specific mAb) and protein G Sepharose at 4 °C for 1.5 h. After three washes with lysis buffer, precipitated integrin was dissolved into 0.5% SDS sample buffer and subjected to nonreducing 7.5% SDS-PAGE and fluorography (Huang and Springer 1997). The efficiency of disulfide bond formation was quantitated using a Storm PhosphorImager after 1 to 3 h of exposure of storage phosphor screens (Molecular Dynamics, Sunnyvale, California, United States). Efficiency was defined as the ratio of the intensity of the disulfide-bonded heterodimer band to the sum of the intensity of all bands including α_IIb_, β_3_, and heterodimer.

#### Two-color ligand binding and flow cytometry

Binding of fluorescein-labeled human fibrinogen was performed as previously described (Pampori et al. 1999; Takagi et al. 2002). To determine the effect of inducible disulfide bond on the ligand binding, oxidation by Cu-phenanthroline was carried out at either 0 °C or 37 °C for 10 min, followed by washing with TBS containing 1 mM Ca^2+^/Mg^2+^ and 5 mM N-ethyl maleimide. Cells were suspended in 20 mM Hepes (pH 7.4), 150 mM NaCl, 5.5 mM glucose, and 1% bovine serum albumin, and incubated with 1 mM Ca^2+^/Mg^2+^ or a combination of 1 mM Mn^2+^ and 10 μg/ml of activating mAb PT25–2. Then cells were incubated with FITC-conjugated fibrinogen with a final concentration of 60 μg/ml at room temperature for 30 min, Cy3-conjugated AP3 was added to a final concentration of 10 μg/ml, and cells were incubated on ice for another 30 min before subjected to flow cytometry. Binding of soluble fibrinogen was determined and expressed as the percentage of mean fluorescence intensity relative to immunofluorescent staining with Cy3-labeled AP3 mAb.

#### LIBS epitope expression

Anti-LIBS mAbs AP5 was from the Fifth International Leukocyte Workshop (Lanza et al. 1994), LIBS-6 was from M. H. Ginsberg, and D3 was from Lisa K. Jennings (Jennings and White 1998). LIBS epitope expression was determined as described previously (Luo et al. 2003). In brief, transiently transfected 293T cells were incubated with either 5 mM Ca^2+^ or 1 mM Mn^2+^ and 100 μM GRGDSP peptide at room temperature for 30 min. Anti-LIBS mAbs (AP5, D3, and LIBS6) was added to a final concentration of 10 μg/ml, and cells were incubated on ice for 30 min before staining with FITC-conjugated antimouse IgG and flow cytometry. LIBS epitope expression was determined and expressed as the percentage of mean fluorescence intensity of anti-LIBS mAbs relative to the conformation-independent mAb AP3 (Luo et al. 2003).

#### Structural model of integrin TM domain at resting state

Model building was performed using the NMR structure of glycophorin A TM dimer (PDB code: 1AFO, model 1) as a template. The entire TM domains of α_IIb_ and β_3_ were aligned, with no gaps. Eighteen different alignments roughly compatible with the observed α–β interface orientation were submitted to the SWISS-MODEL server (Peitsch 1996). For each model, the average Cβ–Cβ or Cβ–Gly Cα atom distance between residues that formed disulfides at greater than 80% efficiency (Trp968–Val696, Val969–Val696, Val971–Leu697, Gly972–Leu697, and Gly972–Val700) was calculated. The alignment where α_IIb_ sequence W968/VLVG and β_3_ sequence V696/LLSV were aligned with glycophorin A sequence G79/VMAG in each monomer gave the lowest root mean square distance (4.8 Å) and thus was chosen as the final model. Models for clusters 11 and 12 were kindly provided by the authors of Gottschalk et al. (2002) and subjected to the same analysis for Cβ–Cβ and Cβ–Gly Cα atom distances.

## Abbreviations

Cu-phenanthroline: Cu(II)-(*o*-phenanthroline)_3_
LIBS: ligand-induced binding site
mAb: monoclonal antibody
NMR: nuclear magnetic resonance
SDS-PAGE: sodium dodecyl sulphate polyacrylamide gel elecrophoresis
TBS: Tris-buffered saline
TM: transmembrane
